# Conference Proceedings for the Fourth International Forum on Quality Cancer Care (IFQCC2020) April 4 and 5, 2020, Abstracts, Queens College, Oxford University, Oxford, UK

**DOI:** 10.36401/i2589-9449-3.5.S1

**Published:** 2020-05-28

## Preface

It is our pleasure to introduce the Proceedings of the Fourth Annual International Forum for Quality Cancer Care (IFQCC2020), which is held at Queens College, Oxford University, Oxford, UK, on April 4 and 5, 2020.

This publication contains abstracts submitted by contributors from seven countries tackling various aspects of cancer care. These abstracts will be presented at the IFQCC meeting in oral and poster sessions to facilitate exchange of experience among participants.

These presentations will be alongside a unique program in which oncology global leaders are discussing critical topics about disparity in cancer care as part of the Cancer Care Commission’s efforts to enhance the quality of cancer care globally.

We would like to thank all our faculty, authors, and staff who contributed to make this event possible.

**Prof. David Kerr**

President, IFQCC 2020

Oxford University, UK

**Prof. Abdul Rahman Jazieh**

Chairman, IFQCC 2020

Ministry of National Guards Health Affairs, KSA

**Abstract Management Committee**

Abdul Rahman Jazieh, David Kerr, Rachel Kerr, Mohammad Alkaiyat, and Valerie Clark

**Organizing Committee**

Calum Kerr and Debbie Stephenson

## ORAL PRESENTATIONS

Considering the Cost of Immune-Related Adverse Events Within the Cost Effectiveness Analyses: A Systematic Review

Fatma Maraiki, Tasneem Elhasan

King Faisal Specialist Hospital & Research Center, Riyadh, Saudi Arabia

**Introduction:** Check point inhibitors (CPI) have demonstrated a wide range efficacy among multiple malignancies but can be associated with costly immune-related adverse events (irAEs). The objective was to evaluate the incidence and trend of reporting the cost of managing the irAEs of CPI, as determined from a systematic literature review for cost-effectiveness analyses (CEA).

**Methods:** Systematic review of all CEA for CPI was identified by a search in electronic databases up to the end of 2018. The review was undertaken to meet the requirements of Preferred Reporting Items for Systematic Reviews and Meta-analyses (PRISMA) reporting method. Data collection included reported total irAEs cost and any other relevant data in how the analysis calculated this cost. Data were collected for analysis with correlation through an SPSS software.

**Results:** A total of 28 published cost-effectiveness analyses, only (46.4%) reported the final total cost of managing irAEs. The cost was mainly considered in higher grades (60.7%) and most common (≥5%) higher incidence (50%), which is not related to irAEs. In total, 80% of the cost reported for irAEs studies were not comprised within the CEA, either not specified or not related to irAEs. Most of the resources were obtained from the literature (46.4%), and around (57.1%) were considered not relevant to immunotherapy by this systematic review author’s assessment. The reported cost was adjusted for inflation in (78.6%) and disutilities were considered in (57.1%) of the analyses.

**Conclusion:** Providing a total cost of irAEs within the CEA were under reported with methodological heterogeneity within the Cost-effectiveness analysis, which warranty a further study.

## Quality of Life in Head & Neck Cancer Survivors After Definitive Treatment -A Single Center Experience

Rajesh K. Agarwal, Zehra Fatima

Royal Cancer Institute, Kanpur, India

**Introduction:** Background: head and neck cancer are the common cancer in northern part of India and incidence is disproportionately high in state of Uttar Pradesh due to high number of tobacco industries and easy availability of tobacco in this part. the purpose of this study to assess the tumor and treatment related factors affecting QOL outcomes of patients received definitive treatment.

**Methods:** in this study 622 patients were evaluated in follow up visit and were asked to fill the EORTC OLQC-30 head & neck module (QLQ H&N 35), and Hospital anxiety score proforma (HAD) developed by Zigmond & Snaith (1983), separately. All questionnaires were filled by patient themselves under observation of a trained psycho-oncologist. Data analysis was done Microsoft Excel. We tried to analyze the worst-case scenario.

**Results:** The compliance rate was high and questionnaire was very well accepted by the patients. Total combined analysis of 622 patients was done. Most of the patients are male (85%),age group is almost equal (48% < 60 years, 525> 60 years.). 85% patients received combined modality of treatment. Only Radiation was in 4% patients. Most common site was oral cavity (71%), mostly of advance stage 58% stage III and IV. Functional scale of HN-35: trouble in long walk 41%; limited in doing daily activity 58%; worried 50%; concentration issue 29%; medical condition effected the family life 50%. Symptomatic scale of HN-30: fatigue felt weak 43%; nausea and vomiting 43%; pain needed rest 34%; dyspnoea 8%; insomnia 19%; appetite 25%; constipation 15%; diarrhea 6%; financial difficulty 49%.

**Conclusion:** The QLQ H&N 35, in conjunction with the QLQ-C30, provides a valuable tool for the assessment of health-related quality of life in clinical studies of H&N cancer patients before, during, and after treatment with radiotherapy, surgery, or chemotherapy.

## Organized Screening for Colorectal Cancer in Algeria: First Pilot Study in North of Africa

Mazouzi Chahira^1^, Bouzid Kamel^2^, Belloul Myriem^1^, Hamdi Cherif Mukhtar^3^, Malha Laoussati^1^

^1^University Hospital Center, Bejaia, Algeria; ^2^Pierre Marie Curie Center, Algiers, Algeria; ^3^University Hospital Center, Setif, Algeria

**Introduction:** Colorectal cancer is a major public health problem in the world. Several studies indicate that it is possible to reduce colorectal cancer mortality by mass screening. In Algeria in 2015, colorectal cancer was the second cause of cancer mortality after lung cancer in men and after breast cancer in women, according to Bejaia’s Registry (2015). It ranks first cancer in men with a standardized incidence of 24 / 100,000 inhabitants and second place among women with a standardized incidence of 16 / 100,000 inhabitants. The increasing CRC incidence and mortality can be reduced by screening and treating adenomas and early cancers. A pilot CRC screening program using immunochemical fecal occult blood testing (iFOBT) and colonoscopy for test-positive were implemented in Béjaia, a northeast district of Algeria, to evaluate the acceptability, feasibility and scaling-up of screening in Algeria. This report describes the implementation, coverage and performance indicators of this pilot project.

**Methods:** This is a pilot study for colorectal cancer screening with an immunological test of an average risk population aged 50–74 years over a 20-month period. A target population aged 50–74 years was informed about and invited to undergo CRC screening by community different-clinic. The referring general doctors provided fecal sample collection kits and participants brought their samples to one of the primary health units where nurses performed iFOBT. iFOBT-positive persons were referred for colonoscopy at the Béjaia University hospital, and endoscopic polypectomy/biopsies were performed according to the colonoscopy findings. Those with confirmed CRC received appropriate treatment.

**Results:** Of the 10,000 target population, 3002 (30.2%) were screened using iFOBT between 2 January 2017 and 31 January 2019. Participation was higher among women than men and lower in 60–74 year-old persons than in 50–54 -year-olds. Of those screened, 210 persons were found positive; positivity was higher in men than in women. 17 cancers were diagnosed so far.

**Conclusion:** The successful implementation of the pilot CRC screening with satisfactory process measures indicates the feasibility of scaling-up organized CRC screening through existing health services In Algeria and North of Africa.

## Evaluation of Adherence to Oral Chemotherapy “Capecitabine”: A Prospective Study from Morocco

Saoussane Kharmoum^1^, Adil Najdi^2^, Saber Boutayeb^3^, Said Afquir^4^, Nawfal Mellas^5^, Hind Mrabti^3^, Fatima Ezzahra El Mrabet^1^, Hassan Errihani^3^

^1^Departement of Medical Oncology, Ahmad Bin Zayed Al Nahyan of Oncology, University Hospital, Tangier, Morocco; ^2^Department of Epidemiology, Faculty of Medicine, Tangier, Morocco; ^3^Department of Medical Oncology, The Research Team in Translational Oncology “EROT,” Faculty of Medicine, Mohamed V university, Rabat, Morocco; ^4^Department of Medical Oncology, University Hospital, Oujda, Morocco; ^5^Department of Medical Oncology, University Hospital Hassan II, Fes, Morocco

**Introduction:** Oral therapies are increasingly developed in oncology, they are a response to a threefold requirement: to improve the quality of life of patients, to give a greater autonomy to the patient, while guaranteeing an equal effectiveness of the treatment. However, these chemotherapies raise new problems, starting with non-adherence to medication, the impact of these therapies on toxicity and quality of life has been little studied, the prescription of oral therapies has become a common practice in oncology, yet little is known about the extent of the variability and diversity of practices by each prescriber. To our knowledge this is the first study in Morocco evaluating adherence of capecitabine.

**Methods:** We conducted a prospective, multicenter, observational study in three teaching hospitals. Patients treated with capecitabine for advanced or early stage cancers were included. The primary endpoint was to evaluate the adherence to capecitabine and quality of life. The secondary endpoints included reasons for the preference of patients for oral treatment, and safety of capecitabine. The adherence was evaluated during three consecutive cycles of capecitabine using indirect assessment methods: self-report and pill count; a patient was considered to be adherent to treatment if he achieved an overall percentage of adherence more than 95% using the two indirect methods. The quality of life was assessed by the EORTC quality of life questionnaire “QLQ 30” In its validated version translated into Moroccan dialect. The practice of medical oncologists was evaluated using a semi-structured questionnaire designed for the study.

**Results:** 125 patients were enrolled between February 2016 and January 2017, the median of age was 51 ± 11,5 years old, 62.4% were male, 71.2% with colorectal cancer, and 19.2% had breast cancer, 60.8% and 37.6% had capecitabine as a first line chemotherapy and in monotherapy respectively. 58.4% of patients preferred oral therapy, the reasons for this preference included: less frequent travelling (52%), avoiding intravenous injection (12%), improved tolerance (14.4%), availability of treatment (4%), better quality of life (1.6%). The detected side effects were mainly of grade 1 and 2. The overall adherence rates were 92% and 90.4% when using self-report, and with pills count respectively. 88% were considered to be adherent (with an overall of adherence > 95% by two indirect methods). Reasons for non-adherence were forgetting to take treatment, and side effects. Factors correlated with non-adherence were living location (p = 0.04) and age (p = 0.07). The involvement of family members was reported in 54.6% of the cases. The overall health score is estimated at 58.88, financial problems were very common, and it was the most affected dimension with a score average of 79.2. Patients with more symptoms tended to be less adherent. 91 medical oncologists were included in the evaluation of the practice of capecitabine prescribing, participation rate among the regional centers practitioners was 100%. Manual prescription forms are still very common (32%), most physician (92.3%) assess adherence through patient interview, 87.9% of them never received specific training in patient therapeutic education.

**Conclusion:** Capecitabine based therapy is associated with a non-neglectable risk of non-adherence. There is a great variability among oncologists in regard of capecitabine in prescribing and follow-up practices. Thus, it’s necessary to promote adherence to oral antineoplastic, mainly by developing local strategies that fit our context.

## Impact of Bed Utilization Management Program on Quality and Cost in a University Oncology Center

Ma Ana Flor Ciocson, Ahmed Abdelwarith, Khalid Alsaleh, Mariam Napuli, Hamoud Ibrahim Alanazi, Irene Abdon

King Saud University Medical City, Riyadh, Saudi Arabia

**Introduction:** The Bed Utilization Management Program (BUMP) was a success story of Medical Oncology Day Care (MODC) in a tertiary-university oncology center in Riyadh, Saudi Arabia. The MODC advanced its operation from six-bed capacity increased to 20 beds, accommodating an increasing number of referred cancer patients. In 2018, the oncology quality improvement team (Onco-QIT) reviewed the occupancy rate (87%) and perceived as below optimum bed utilization despite a 300% increase in bed-capacity. Many factors were identified through root cause analysis (e.g., delay in chemotherapy schedules, unnecessary chemotherapy inpatient admissions, no clear bed management pathway, and a high rate of no show on appointment (NSoA) (15%). The aim was to achieve 100% optimum bed utilization through BUMP within six months that will impact on lowering the rate of high-cost unnecessary patient’s admission.

**Methods:** The Onco-QIT selected the FOCUS-PDCA model. Its nine steps stand for F = find a problem, O = organize a team, C = clarify the problem, U = understand a problem, S = select an intervention, P = plan, D = do, S = study, and A = act. The sources of data collection were key performance indicators on bed occupancy rate and no show on appointment.

**Result:** There were two positive outcome results of this quality improvement initiative. The average bed occupancy rate was 98%, and NSoA was 7% within three quarters. The BUMP consists of comprehensive strategies and interventions that were measurable by outcome indicators. First, the Onco-QIT developed and implemented a bed management pathway to improve bed utilization in MODC from pre-admission, admission, bed management, and discharge/transfer. Second, the center hired an oncology case manager is an integral part of bed management in triaging and allocating the patient’s bed on admission. The case manager increases the frequency of bed utilization for short term and intermediate chemotherapy beds more than once per day. Third, the Pharmacy interventions consist of compliance of single-agent and long term protocol guidelines, periodic communication of unavailable chemotherapy drug list to MODC, and acquiring new oncology satellite pharmacy. Fourth, there was a significant reduction of No Show in MODC after the implementation of a reminder system by the admission clerk via mobile messages or calls to the patient for the appointment confirmation. Since the program is a continuous dynamic development, all nurses were chemotherapy certified. Lastly, the OQT developed five policies based on the accreditation standard of cancer care services.

**Conclusion:** Overall, the implementation of the bed utilization management program is vital in managing cancer patients. It minimizes the high rate of admission cost and maintains the chemotherapy time table plan of the patient.

## Trends of Patients Perception About Cancer Causes and Risk Factors Over Time

Mohammad Alkaiyat^1^, Khadega Ahmed^1^, Hussam Ardah^2^, Abdul Rahman Jazieh^1^

^1^Department of Oncology, King Abdulaziz Medical City, Ministry of National Guard Health Affairs, Riyadh, KSA; ^2^King Abdullah International Medical Research Center, Ministry of National Guards Health Affairs, Riyadh, KSA

**Introduction:** Patient perception and prevalent myths about cancer are very important to recognize as they may interfere with cancer management and control efforts. In this study, we evaluated patient’s perceptions about cancer causes and risk factors and its evolution over time.

**Methods:** Patients with cancer were enrolled in a cross-sectional study about the perception of cancer and the use of complementary and alternative medicine (CAM). The patients were enrolled between 2006 and 2018 in two cohorts. Cohort 1 enrolled between 2006 and 2008 and cohort 2 were enrolled between 2016 and 2018. The perception about causes of cancer and cancer prognosis were obtained and analyzed with comparison between two cohorts of patients.

**Results:** A total of 1416 patients were enrolled in the two cohorts (Cohort 1 N = 464 patients and cohort 2 N = 952). The two cohorts matched in age and gender. 224 patients (23.5%) of cohort 2 gave scientific causes for cancer compared to 63 (13.6%) of cohort 2. Females were more likely to give mythical causes of cancer. There was significant increase in the belief of evil eye (1.29% to 33.09%), evil spirit (0.2% to 5.15%) and reduction in jealousy (29.74% to 5.57%) from Cohort 1 to Cohort 2. Considering smoking as cancer risk increased from 1.29 % to 4.52% only.

**Conclusion:** Our study revealed common misperceptions about cancer that require systemic approaches for patients and public education to minimize any negative impact of these misconceptions on patient’s management, quality of life, and outcome.

## POSTER PRESENTATIONS

Poly-pharmacy in Patients with Cancer: Need for Robust Primary Care During Cancer Treatment

Mohammed Saeed, Ihab Sharha, Mohammad Alkaiyat

Department of Oncology, King Abdulaziz Medical City, Ministry of National Guard Health Affairs, Riyadh, KSA

**Introduction:** Due to the lack of robust primary care, many patients with cancer and comorbidities or chronic diseases continue to take several therapeutics prescribed for different reasons by non-oncology-hematology medical specialists and resulting in iatrogenic serious side effects. It demands higher level of vigilance due to narrow safety window in this special segment of patients. These include medications for high blood pressure, diabetic medications, over the counter painkillers, most importantly, NSAIDs and use of combination lipid lowering medications.

**Methods:** In our retrospective analysis of a cohort of 100 patients who had primary diagnosis of cancer, we found high proportions of patients taking several different therapeutics which were not indicated, labeled as futile management and indeed having potential harm to patients. This phenomenon of poly-pharmacy was observed in last 60 days of life.

**Results:** 41% of patients were taking 10 more medications, 35% were taking 6–9 medications, and 16% were taking up to 5 medications. Only 2% were taking 1–4 medications. 59 diabetic patients have documented clinical hypoglycemia while on diabetic agents. Other clinical findings were hypotension, dehydration, bleeding, acute renal damage, and falls. Most common medications were anti-hypertensive, diuretics, diabetic medication, over the counter pain medications, narcotics, and anticoagulants.

**Conclusion:** In our cohort of 100 patients, there is an alarming prevalence of poly-pharmacy in last 60 days of life, our finding is tip of the iceberg for this widespread quality of care issue. Due to fragmented presence of primary care in geographical diverse areas of Saudi Arabia, elderly patients with cancer are bearing the most burdens and significant side effects related to absence of this robust primary care oversight during the course of cancer treatment. About 15% of patients who have emergency room visits and eventual admissions to hospital, were found to have potential side effects of these non-indicated therapeutics.

## Improving Coordination of Lung Cancer Care at Ministry of National Guard Health Affairs – Central Region

Abeer Alkhathlan^1^, Razan Alfaiz^1^, Ghaida Almusallam^1^, Esraa Arabi^1^, Mohammad Alkaiyt^2^, Abdul Rahman Jazieh^2^

^1^College of Medicine, King Saud bin Abdulaziz University for Health Sciences, Ministry of National Guard Health Affairs, Riyadh, KSA; ^2^Department of Oncology, King Abdulaziz Medical City, Ministry of National Guard Health Affairs, Riyadh, KSA

**Introduction:** The improvement of healthcare outcomes is the ultimate aim for any health institution. Coordination of care is very critical for providing safe and efficient care. The aim of this study is to improve the care coordination for cancer care which involves patients flow in the healthcare system and providing care that is safe, timely, efficient, effective, and patient-centered.

**Methods:** We conducted a prospective study to collect data to be used as baseline for a rapid cycle of improvement PDSA (plan-do-study-act) project. We began with collecting and analyzing data for lung cancer patients who were diagnosed in 2016/2017, collecting information about their demographics, the interval between suspecting cancer and confirming diagnosis, the interval between cancer diagnosis and receiving definitive cancer therapy, tumor board (TB) data (presentation, adherence to recommendations, TB compliance), and palliative care.

**Results:** A total of 60 cases of lung cancer were evaluated in 41 males and 19 females. The majority of these cases had adenocarcinoma (63.3%) and squamous cell carcinoma (23.3%). The stages of the tumors were as follows: 42 stage IV (70%), 7 stage II (11.7%), 6 stage III (10%), 2 stage I (3.3%), and 3 stages were missing (5%). Adherence to guideline of EGFR testing was 100%, while it was 82% for ALK, and 71.4% for ROS1. PDL-1 testing was done for 59.5% in stage IV patients. The median number of days from first visit to oncologist and the palliative care referral is 35 days [0–643]. Specifically, for stage IV patients, 32 (76.2%) were referred to palliative care. For presentation in tumor board, 40 (66.7%) of all patients had presented in the tumor board, some of these cases presented multiple times; the total presentation in tumor board is 65 times. Of these presentations, 31 (51.7%) were before treatment and 9 (15%) were after. The presentation of these cases revealed new findings in pathology 3 (7.5%), in radiology 7 (17.5%) and 5 (12.5%) had stage related new findings. In total, these new findings appeared in 11 (27.5%) unique cases. Tumor board discussion had shown an impact in 14 (37.5%) out of 40 patients presented, while the impact reduced to 14 (21.5%) out of 65 for each time the patient were presented in tumor board.

**Conclusion:** The molecular testing for actionable targets beyond EGFR was limited by the inadequate tissue which was minimized by the implementation of next generation sequencing (NGS). Better processes for referral to palliative care and tumor board presentation are being implemented.

## Choosing Wisely by Implementing Early Complex Advance Care Planning to Reduce Burden of Futile Management at the End of Life and Improve Quality of Life for Patients and Caregivers

Mohammed Saeed, Ihab Sharha, Mohammad Alkaiyat

Department of Oncology, King Abdulaziz Medical City, Ministry of National Guard Health Affairs, Riyadh, KSA

**Introduction:** Cancer-related terminal illness brings many uncertainties for patients and families, which further creates enormous psycho-social, physical, and existential burden, and it is of an imperative value to learn and implement complex advance care planning via team approach in all metastatic cancer patients on early basis.

**Methods:** We conducted a retrospective analysis of a cohort of 103 patients who died in the palliative care unit during the year 2018. There were 43 males and 60 females, among them 101 of Saudi citizens and 2 patients were other nationalities.

**Results:** In this cohort, advance care planning was made during last few weeks of life. Our data analysis revealed that 66% (68 patients) died within 30 days, among them 46.6% (48 patients) within 14 days, 28% (28 patients) within 7 days, 14.5% (15 patients) within 3 days, and 4% (4 patients) died during same day of transfer to palliative care service.

**Conclusion:** Complex advance care planning was made quite late in these terminally sick patients with advance malignancies, which resulted in tremendous burden of futile management and poor quality of life for patients and caregivers.

In a recent study published online on Nov.16 in *JAMA Oncology*, several quality indicators were identified which have highest potential to reduce spending without compromising high-quality oncology care, one of the quality indicators was “early discussion of limitations and consequences of treatment” i.e., advance planning.

## Cost Analysis of a Hospital-Based Palliative Care Program for Patients with Cancer

Mohammed Saeed, Ihab Sharha, Mohammad Alkaiyat, Abdulaah Al-Qarni, Abdul Rahman Jazieh

Department of Oncology, King Abdulaziz Medical City, Ministry of National Guard Health Affairs, Riyadh, KSA

**Introduction:** The palliative care needs are on the rise across Gulf countries with economical and quality of health-care implications. Our study aimed at conducting cost analysis of a hospital-based palliative care program for patients with cancer in tertiary cancer hospital in Saudi Arabia.

**Methods:** Retrospectively, we collected the data of patients who passed away in the palliative section of the oncology department in 2017–2018. Using standardized data collection tool that focusses on the patients’ resources utilization science their transfer to palliative care.

**Results:** We collected the data of 103 patients who died in palliative care unit (PCU) during the study period. Mean length of stay in PCU was 18 days. 73.5% of patients had at least one invasive procedure, 20.6% of patients had two procedures, 2.9% had three invasive procedures and another 2.9% had up to six invasive procedures. 82.5% visited ER one time, 15.5% have two ER visits, and 1.9% has 3 ER visits. Substantial number of patients were transferred to PCU in just 10 days before death. 80.6% of patients have no outpatient visit after enrollment to Palliative Care Services while remaining patients visited only one time to outpatient palliative care. Complex advance care planning was made during last few weeks of life. Most patients have multiple daily labs and radiological investigations.

**Conclusion:** Palliative care is very much needed nowadays across Saudi Arabia, and having cost-effective care requires step-down programs. Early complex advance care planning is an essential tool to combat futile management. Deaths in hospital may reflect limitations for palliative care providers for alternate placements. Current evidence for high prevalence of futile management at the end of life would increase the cost of palliative care program sky high while having no evidence of potential benefits in terms of reduction of morbidity and mortality and would also increase poor quality of life for terminally ill patients with cancer and their caregivers.

## Prevalence of Futile Acute Care Interventions for Patients with Advanced Cancer: Impact of Delayed Documentation of Goal of Care

Hind Salama, Nashmia AL Mutairi, Ashwaq Alolayan, Ahmed Binahmed, Hagir Salama, Mona Shami, Hussam Shehata, Mohammad Alkaiyat, Abdul Rahman Jazieh

Department of Oncology, King Abdulaziz Medical City, Ministry of National Guard Health Affairs, Riyadh, KSA

**Introduction:** Documentation of goals of care for patients with advanced cancer is known to be poor worldwide. At King Abdulaziz Medical City Oncology Center, we observed that many patients with advanced cancer undergo critical care interventions which have limited benefit, more cost and stress to patients, families, and providers. The main reason for that is lack of timely decision and documentation of goal of care. Our quality improvement project aims to assess the magnitude of the problem in order to improve the practice.

**Methods:** A multidisciplinary team reviewed retrospectively the records of cancer patients who died in hospital at our department from November 2017 until May 2018. The collected data included stage, type of cancer, aim of therapy, causes of death, need for critical care interventions and the time of documentation of goal of care. Our definition for the timely documentation of goal of care is the availability of electronic documentation for code status before the patient needs critical care response team (CCRT), code blue or intensive care unit (ICU) admission.

**Results:** A total of 161 case were reviewed, 90% of the patients had advanced cancer, 90% died due to disease progression; however, only 52.8% had timely goal of care documentation and 26.7% of them died in ICU. The intent of treatment was palliative for 83% (n = 135). Analysis of those 135 cases showed that the goal of care was timely documented in only 59.3% of them and 31.4% died within 7 days of the goal of care documentation, 25.2% and 31.9% of them had CCRT activation and ICU admissions, respectively. 70.4% were referred to palliative care, 64.2% of those referred to palliative care died within 30 days of referral.

**Conclusion:** Delayed goal of care documentation resulted in improper utilization of ICU beds, intubations, and mechanical ventilation. Interventions to reduce this futile practice are underway.

## Straight to CT: A Review of the Lung Cancer Diagnostic Pathway in Patients with Suspected Lung Cancer

Alexander Sheeka, Susannah Bloch

St. Marys Hospital, London, UK

**Introduction:** The National Optimal Lung Cancer Pathway (NOLCP) is the current gold standard for investigating patients with suspected lung cancer. As part of the pathway, a 6-day time-limit is recommended from acquisition of a chest x-ray to patient attendance in clinic with a reported chest CT-Scan. The purpose of this paper is to compare the lung cancer diagnostic pathway at our hospital to the set NOLCP standard.

**Methods:** The clinical records of 55 patients (33 outpatients, 22 inpatients) referred through the lung cancer pathway between September and December 2018 were reviewed. Data was acquired regarding the time from initial x-ray acquisition to reporting, time from x-ray reporting to CT chest acquisition, and the total time from x-ray acquisition to CT chest reporting.

**Results:** Of the 33 outpatients median time from x-ray acquisition to report was 12.6 hours, from x-ray report to CT completion was 173 hours, and x-ray report to CT report was 213 hours. Of the 22 inpatients median time from x-ray acquisition to report was 18 hours, from x-ray report to CT completion was 215 hours, and x-ray report to CT report was 317 hours.

**Conclusion:** Based on this preliminary review the total time from lung cancer suspicion to CT reporting is significantly longer than the currently recommended national guidelines. As a result, funding has been given to triple the dedicated lung cancer CT scanning slots at our hospital trust. Currently data is being collected for a re-audit to assess the necessary number of scanning slots to achieve the NOLCP guideline time.

## Liquid Chromatography-Mass Spectrometry Analysis for Measurement of Acetate in Pharmaceutical Peptide

Rani Qassem, Eman Alshmmeri, Lubna Alhydan, Atheer Alkeraidees, Amjad Abdullah

King Saud bin Abdulaziz University for Health Sciences, Riyadh, KSA

**Introduction:** Liquid chromatography–mass spectrometry (LC-MS) is a novel method which can measure the acetate content in different pharmaceutical peptides, as well as amino acids sequence, mass, and purity. The objective in this study is to assess the sensitivity and specificity of the LC-MS/MS method in comparison to high-performance liquid chromatography (HPLC) method in measuring the acetate content in cetrorelix, which is the drug used as synthetic deca-peptide with gonadotropin-releasing hormone (GnRH) antagonist effect.

**Methods:** We studied the acetate content in injectable cetrorelix by the two methods (HPLC and LC-MS/MS). In LC-MS/MS method, cetrorelix was the active pharmaceutical ingredient (API) and formic acid was the solvent. The method uses gases to run the experiment with added C13 labeled acetate. 12 samples were prepared containing 10 ug/mL C13 and 1 mL C12 acetate with different API concentrations. LC-MS/MS method require to switch the ionized mode into a negative mode in order to measure the acetate content, then switch it to positive mode in order to detect other impurities.

**Results:** In the LC-MS/MS method average concentration for acetate is 0.0810 and 0.0836 in milligrams per vails for all samples, which were similar in both methods with recovery percentage accuracy 98.8%. The method was linear for acetate concentrations with a coefficient of determination (*r*2) equal to 0. The relative error of the calculated concentrations and actual ones were less than 5% for all reading. According to the chromatogram, the drug purity is 95%. In addition, the calculated molecular weight was equal to 1429.5 g/mol. Acetate peak retention time was 7.5 min using the HPLC method compared with 13 minutes using the LC-MS/MS.

**Conclusion:** LC-MS/MS method is multiplexed method that help collecting quality criteria required in regulatory submissions such as the Food and Drug Administration in a single experimental setting. As a result, this helps in minimizing the cost and save time and effort.

## The Journey to Maintain Zero Adverse Events in Stereotactic Radiosurgery Program by Process Reengineering

Salem M Alshehri, Khalid Alsurimi, Abdulrahman Alhadab, Saif Althaqfi, Rania Ali, Mamdouh Alqathami, Ahamed Yoosuf, Mohammad Alkaiyat, Abdullah Alzahrani, Abdul Rahman Jazieh

Department of Oncology, King Abdulaziz Medical City, Ministry of National Guard Health Affairs, Riyadh, KSA

**Introduction:** The main principle of SRS is delivering a precise and accurate high dose of radiation to metastatic cancer site(s) or lesion(s) with sparing the normal structures. Once the radiosurgery dose is delivered it cannot be reversed and the effect is permanent. The SRS dose is quite variable with complex delivery process. It involves several team members from completely different units, e.g., physicians, medical physicist, planner, and therapist, which necessitates several safety checks done by physicians and nonphysicians. There is no outlined standard procedure to be followed and communicates all team members to deliver the prescribed radiosurgery dose. Electronic Treatment Directives (TDs) will enhance patient’s safety and eliminate the chances of error in administering SRS dose. These electronic treatment directives are expected to improve our department’s workflow, effectiveness, and efficiency. These electronic TDs will serve as a standard path for each type of case in the belief that standard work leads to enhance safety, more effective communication, more efficient use of resources and more easily allow specification of non-standard instructions.

**Methods:** Root Cause Analysis and Process Mapping. Reporting (count of Adverse events). Rounding compliance report to (TDs) upon patient assessment in clinic. Average processing time (Turnaround) simulation to treatment time. Patients volume treated with SRS in department. Staff satisfaction survey. Run chart, Control chart, Xbar-S and segmented regression charts were used to analyze and interpret data.

**Results:** Adverse Events were maintained at zero level. Compliance to electronic (TDs) increase to 80% in February 2019 then peaked to 100% by March2019 and sustained till Dec. 2019. While increasing in patient volume, the mean average processing time (Turnaround) simulation to treatment time decreased from 13.56 days (pre-TDs) to 9.95 days (post-TDs) which reflects increase in efficiency and cost effectiveness.

**Conclusion:** Standardizing pathways of prescribing SRS enhance patient safety. Improvement of communication increase efficiency. Understanding the data and analyzing them in scientific approach leads to improvement. Involvement of front-line staff in improvement reflects positively to project success.

## Dosage Adjustment for Cytotoxic Drugs: Guidance or Confusion

Ersan Alzamel

Milton Keynes University Hospital, Milton Keynes, UK

**Introduction:** Organ dysfunction in cancer patients is common. It poses a dilemma for the treating physicians who should tailor the doses while prescribing chemotherapy. Caution is compulsory since most cytotoxic drugs exhibit a narrow therapeutic index; this implies that an inappropriate reduction in dose may lead to undertreatment of patients. In contrast, the absence of dose reduction, when clinically indicated, may lead to excessive toxicity.

**Methods:** The published dosing recommendations are available on the networks. They were reviewed and compared to find out their shortcomings and the strategies in which the physician-in-charge are using in daily practice.

**Results:** Several shortcomings were found: They are largely empiric. They are frequently based on case studies or retrospective observations in small numbers of non-homogeneous patients. There is paucity of formal Phase I studies on pharmacokinetics and clinical toxicity. Most of the time, organ-impaired patients have been excluded from clinical trials. They are derived from clinical data that are decades old and preceding the routine use of supportive drugs, e.g., colony-stimulating factors. There is inconsistency in the definitions used for organ dysfunction, e.g., there is no standardized system with which to define liver or renal dysfunction. In oncology drug labels, the statement insufficient data available in renal and hepatic impaired patients, caution should be exercised presents a subjective approach rather than personalized medicine for cancer patients. Different sources show high variability in dose recommendations interfering with adequate dose adjustment and causing high level of confusion. To overcome the aforementioned problems, several strategies were applied: o Individually adjusted dosing according to patient, tumour and genotypic characteristics. o Selection of drugs which are less dependent on organ dysfunction. These safe options serve as a bridge to more conventional chemotherapy. o Starting with dose reduction of chemotherapy empirically with re-escalation of dose in the absence of dose-limited toxicities. o Altering a drug schedule by avoidance of combination therapy, divided doses or prolonging the duration. o Treating patients with chemosensitive tumors, e.g., breast cancer, germ cell tumors and lymphoma, who have tumor-related organ impairment at the recommended doses.

**Conclusion:** In the absence of reliable dose-modification schemes, empirical guidelines are still largely applied in clinical practice. From a national and international perspective, the following prerequisites are needed: A formal dose-escalation Phase I study with a complete pharmacokinetic profile as the primary end point and toxicity as the secondary end point should be conducted. Establishing a national network for individual dose adjustment in cancer patients undergoing chemotherapy and experiencing organ dysfunction. Extrapolate the extravasation Green Card Scheme or the yellow card scheme of Medicine and Healthcare Regulatory agency (MHRA) to create the organ dysfunction reporting scheme.

## Medication Early Release Program

Mohsen Al Zahrani, Myer Lawrence, Mohammad Al Harbi, Mona Al Shami, Houssam Shehata

Department of Oncology, King Abdulaziz Medical City, Ministry of National Guard Health Affairs, Riyadh, KSA

**Introduction:** The outpatient oncology infusion suite is a very busy unit and sees an average of 60 to 70 patients per day. A limited number of treatment chairs, one pharmacy hood for biohazardous drug preparation and various other factors have resulted in long waiting times for patients before starting their treatment. To improve the patient experience and to try and alleviate pressure on the preparation pharmacy and nursing services during peak times we investigated the feasibility of an early medication preparation and release program. This would enable rapid admission, assessment and administration of the pre-prepared treatment and in turn have a positive impact on patient flow and equalize the pressure felt by the preparation pharmacy.

**Methods:** In this quality improvement project we formed a committee that met on biweekly basis, this committee included representatives from nursing, quality, pharmacy, data analysis and was led by a consultant physician. We studied the baseline data of patient waiting times from January to March 2019 and factors that caused patient delays in being treated. Patient selection criteria were prepared to identify patients that could safely have their medication released early in the morning at 7 am enabling pharmacy to dispense at 8 am without their actual presence being required in the infusion suite. Multiple PDSA (plan-do-study-act) cycles with several process changes and educational interventions were implemented to achieve our goal. The data collected included check-in time, chair time, vital signs time, administration time and discharge time, finally we collected data on any patients who did not receive medication that had already been prepared which resulted in drug wastage and the reasons why.

**Results:** Baseline data identified that the average waiting time for patients receiving similar medications to those identified in the medication early release program (MERP) was 2.27 hrs a with a range of waiting times between 33 minutes to 5.07 hrs. After the first intervention the average waiting time reduced to 1 hr and 24 minutes through each PDSA cycle we could see small improvements in the average time. The major breakthrough appeared following the intensive patient education program and enforcement of strict compliance with the criteria in selecting patients appropriate for the early release program. We reached our goal of 1 hr in the 8th month of the programs. We identified drug wastage for patients who did not show up for treatment as a balancing measure. We were successful in reducing the drug wastage by applying several changes and patient education measures.

**Conclusion:** A positive impact has been seen with a significant reduction in the average waiting time from 2.24 hours to 1 hour exactly. With further interventions relating to patient education and root cause analysis of any patients who are spending longer than necessary in the waiting area we aim to improve this further, an expansion of the pharmacy′s capacity to prepare more than 10–15 preparations between 7–8 am may give rise to a further expansion of the criteria for patients to be included in the MERP pathway.

## Percentage and Prognostic Significance of *ERCC1*, *KRAS*, *BRAF* in Colorectal Cancer in Egyptian Patients

Maha Saif^1^, Sameh Shamaa^1^, Mohamed Awad^1^, Mie Ali^1^, Hayam Fathey^1^, Abdel Rahman Rouda^2^

^1^Oncology Center, Faculty of Medicine, Mansoura University, Egypt; ^2^Pathology Department, Mansoura University, Egypt

**Introduction:** *ERCC1* is the gene that involved in nucleotide excision and DNA repair. It was discovered a strong relation between *ERCC1* expression and the response of the patients with metastatic colorectal cancer (CRC) to FOLFOX regimen. *KRAS* is a GTPase and is an early player in many signal transduction pathways *KRAS* mutation prognostic significance was in question, it was revealed that KRAS may be prognostic for CRC patients. *BRAF* is a human gene that produces a protein called BRAF which is responsible for cell signal transduction. In CRC, BRAF is a poor prognostic factor in numerous retrospective assessments. This study aimed to evaluate the prognostic and predictive value of *ERCC1*, *KRAS,* and *BRAF* mutations in CRC by IHC method.

**Methods:** This was a retrospective study of 181 patients with CRC stage I to IV in which tissue microarray blocks from 181 formalin-fixed paraffin embedded colorectal tissue blocks were collected from the Oncology & Gastroenterology Center of Mansoura University from January 2006 to December 2014. IHC staining with monoclonal mouse antibody specific for *ERCC1*, *KRAS*, and *BRAF* were done, and expression was scored for *ERCC1* by H- score, which is calculated by multiplying the intensity and percentage of *ERCC1* positive cells: *ERCC1*-positive patients (H score ≥ 2), *ERCC1*-negative patients (H score < 2). Also, expression was scored for *KRAS*-positive (if *KRAS* staining in ≥ 10% of cells), *KRAS*-negative (staining in < 10% of cells), and for *BRAF-*positive (*BRAF* staining in ≥ 10% of cells), *BRAF*-negative (staining in < 10% of cells).

**Results:** One hundred and four patients with H score < 2 were negative for *ERCC1* (57.5%) and 77 patients with H score ≥ 2 were positive for *ERCC1* (42.5%). Disease-free survival (DFS) showed no difference between *ERCC1*-negative and positive cases. The median progression-free survival (PFS) for patients receiving oxaliplatin-based chemotherapy in the metastatic setting is higher in *ERCC1*-negative cases (median: 11 months, 95% CI: 7–14 months) than *ERCC1*-positive cases, and overall survival (OS) showed no difference between *ERCC1*-negative and positive cases. *KRAS* was positive in 89 patients (49.2%) and negative in 92 patients (50.8%). The DFS showed no difference between *KRAS*-negative and positive cases, and the median OS is significantly prolonged in *KRAS*-negative cases. *BRAF* was positive in 46 patients (25.4%), which is higher percentage in Egyptian patient than other patients, and negative 135 patients (74.6%). The 3 and 5 years DFS is higher in *BRAF*-negative cases. However, the difference is not statistically significant, and the median OS is significantly longer in *BRAF*-negative vs. positive cases.

**Conclusion:** *ERCC1* is predictive factor for response to oxaliplatin and PFS in metastatic diseases. *BRAF* may have higher percentage among Egyptian patients (25%), and *BRAF* score is a prognostic factor of DFS. *KRAS* and *BRAF* are prognostic factors for OS.

## Hematological and Digestive Toxicity Profile of Platinum Salts

Zoubir Derbouz, Aimene Melzi, Sarah Henni Manseur, Nora Kessi, Adda Bounedjar

Blida University Hospital, Blida, Algeria

**Introduction:** The toxicity of platinum salts is a factor that can hinder the proper use of treatment and put at risk the vital prognosis of patients including hematological digestive and toxicity. Given the absence of this type of national clinical research, we took this initiative to describe the profile of the hematological and digestive toxicity of platinum salts in the Algerian population. The purpose is to describe and evaluate the hematological and digestive toxicity profile of platinum salts (cisplatin, carboplatin, and oxaliplatin), both biologically and clinically.

**Methods:** This is a prospective descriptive study, which was conducted at the medical oncology department of Blida University Hospital between January 2017 and May 2017. We included patients receiving one of the three platinum salt molecules. The evaluation of different grades of toxicity was made based on the World Health Organization (WHO) toxicity rating table. The collection and analysis of the data was performed in Microsoft Excel 2013.

**Results:** We included 72 patients (40 patients are evaluable). The mean age is 53 yrs, 27.80% and 26.40% of patients were aged 50–59 and 60–69, respectively, and 27.80% and 26.40% with predominant use of carboplatin. More than half of the patients received carboplatin, while cisplatin and oxaliplatin were used in (23.6%) and (20.8%) cases, respectively. The lungs and head and neck are the most common sites (25%) with predominant use of carboplatin for the lung, and cisplatin for head and neck. Patients diagnosed at the late stage (III and IV) represent 82 % of cases with preferential use of carboplatin. The mean rate of leukocyte depletion is about 28% for the platinum salt class. A decrease of half is observed in patients under cisplatin and oxaliplatin, and of 10% for carboplatin. A decrease of neutrophils of 29% is noted with the class of platinum salts. It is more marked for oxaliplatin and cisplatin (41% and 42%, respectively) compared to carboplatin (19%). The depth of decrease in hemoglobin between the baseline and the fourth cycle is greater for cisplatin (15%) and carboplatin (12%) than for oxaliplatin (6%). The platelet reduction rate variation between the baseline and the fourth cycle with oxaliplatin and carboplatin is 16% and 14% respectively. A slight increase of 2% is observed with Cisplatin. About 20% of patients had Grade I or II leukopenia and neutropenia, and only 10% had Grade III or IV. Cytopenic grades I and II are similar for all three molecules, but no grade III and IV toxicity with cisplatin. Grade I and II anemia is very common with carboplatin (31%) and oxaliplatin (19%) and rare with cisplatin, whereas severe grade III and IV anemia is exclusive for carboplatin. The digestive toxicity of platinum salts is more pronounced with carboplatin and oxaliplatin. More than half of the patients have CINV (chemo-induced nausea and vomiting), 44% diarrhea, and 34% constipation (all grades combined). Grade I and II mucositis and stomatitis are very common with carboplatin while Grade III and IV stomatitis are observed only with oxaliplatin and cisplatin (3% and 1%). Grade I and II nausea and vomiting are present with all molecules at the same frequency, while grades III and IV are more marked with carboplatin.

**Conclusion:** A carboplatin is the most hematotoxic molecule with an occurrence frequency of 8% leukopenia, 12% neutropenia, 2% thrombocytopenia, and 33% anemia. Cases of thrombocytopenia have been observed exclusively with carboplatin with a frequency of 2% (grade I and II). It is estimated that there is more than 40% incidence of mucositis according to the literature. For our population, the incidence is 65%.

## Current Status of Risk-Sharing Agreements in Portuguese Health Care System: Preliminary Results

Ana-Marta Silva, Luís Filipe Azevedo, Francisco Rocha-Gonçalves

Faculty of Medicine of the University of Porto, Porto, Portugal

**Introduction:** Risk-sharing agreements (RSA) are typically implemented to manage uncertainty related to clinical benefits and cost-effectiveness in the real world. Thus, they allow the patient to have faster access to innovative medicines since there is a financial risk sharing between payer and the pharmaceutical industry. This type of agreements is used internationally in cancer medicines, as this is a constantly developing area. Gonçalves FR et al. (2018), mention that in Portugal, 40% of the RSA are in Oncology. This work aims: (1) to assess the knowledge and experience about the RSA from professionals who work in the Portuguese Health Care System; (2) to determine the experience of Health Institutions with this type of agreements; and (3) to identify the barriers that exist for the application of the RSA.

**Methods:** A literature review was performed to develop the questionnaire to apply in our study. Afterwards the questionnaire was refined with the help of experts in the area and a pre-test was performed. This questionnaire will be sent to all Portuguese hospitals in February 2020 via a Google form and will be available for 1 month. It will be addressed to hospital administrators, hospital management staff and hospital pharmacists. An adapted version of the questionnaire will also be sent to the National Authority of Medicines and Health Products, I.P (INFARMED).

**Results:** We expected to observe that Portuguese Health Care System professionals are familiar with the RSA. We also target to quantify the number of RSA in Portugal and to classify their type. We think the results will show that RSA are not being used in most of the Health Institutions, although surveyed professionals recognize the potential value in their use. The two biggest barriers we expect to find in the application of RSA are the lack of human resources and computer systems that allow quick and simple information acquisition and assessment.

**Conclusion:** The questionnaire will be very useful for gathering information on the topic of RSA and understanding the perception of the healthcare system professionals in Portugal, particularly regarding the application of RSA. It will also increase understanding about barriers/challenges that this type of agreement face, allowing the development of strategies to improve their use.

## Access to Cancer Therapeutics in Private Practice Setting in Algeria

Houda Jamous, Nezha Benharrats, Aicha Ikash

The Hope Oncopole Center, Oran, Algeria

**Introduction:** Being the second cause of mortality and considering its incidence and the cost of its management which are evolving in an exponential way, cancer becomes a real public health problem in Algeria. Although the Algerian government finances cancer management with billions of dinars, it still cannot offer to all citizens suitable healthcare services regularly and continually. This reason opened timidly the doors to private clinics to alleviate the load and to facilitate and accelerate patients’ access to care in better circumstances. The Hope Oncopole, Hope Cancer Care Center (CCC), Oran, Algeria, is a private cancer care center which started offering its services of radiation therapy about 2 years ago and chemotherapy from a few months ago. This study aims to describe the access of Algerians to private cancer care and cancer therapy, and the resources used and impact on patients.

**Methods:** A prospective study of all patients from July 2019 to December 2019, in medical oncology Department, Hope CCC, Oran, Algeria. Inclusion criteria: age ≥ 18 years, patients who presented more than once to oncology consultation for the management of a solid cancer disease with a chemotherapy indication.

**Results:** During the 6 months, we received 146 patients, 50 were excluded (37 who never came back after the first consultation, 4 pediatric cases, 4 hematologic malignancies, 5 no actual indication of chemotherapy). We prescribed chemotherapy to 96 patients, male‐female ratio 0.4, median age 59 years (19–86), 57% from Oran, 43% from surrounding cities. 29% presented with breast cancer, 23% with digestive cancer, 17% head and neck cancer, 14% gynecologic cancers, 7% thoracic malignancies, and 5% GU cancer, 5% others. 45% had a metastatic disease, 21% came for concomitant chemoradiation therapy. 42% had chemotherapy in our center, 25% paid with their own means, while 2% got the family financial support to get the 3 first cycles of treatment, after they got an objective response they moved to public hospital, 2% sold their cars and 2% sold some jewelry to settle the account, 4% had cancer patients support organizations funding, the remaining 15% had no financial issues. 17% had employees who covered their healthcare fees. Among these patients: 16% finished the total of their treatment, 20% are still having their chemotherapy, 2% died because of their cancer, 2% could not afford to pay the rest of their cycles, 1% had 1 cycle because she was in Oran just for holidays, and 1% decided to interrupt her treatment in order to start a phytotherapy. 2% were working while being treated, 19% stopped working, 19% were retiree, and 60% were jobless. 58% hadn’t chemotherapy, 3% died by their cancer, 13% were unfit, 3% refused chemotherapy, 27% because of lack of material went to public hospitals, 12% were lost of follow up.

**Conclusion:** Systemic therapy is an essential cancer treatment modality. Although private sector may offer cancer patients easier access to treatment, unfortunately the cost is a critical barrier to care for the majority of patients in Algeria. Family and charity support can sometimes help patients to cover part of the treatment. Health insurance is not participating yet in covering cancer care in significant way.

## Prescription Rate of Systemic Anticancer Therapy in the End of Life Situation (at 60 days or Less) in Terminally Ill Hospitalized Oncology Patients: Single Center Experience

Ahmed Ayyad, Hatoon Bakhraibah, Abdullah Altwairqi, Abdullah Alsharm

King Fahad Medical City, Riyadh, KSA

**Introduction:** Discontinuation of anti‐cancer therapy is recommended in terminally ill cancer patients(pts) who are determined to be not fit. Persistence use of aggressive therapy in patients who deemed unresponsive to cancer therapy can negatively affects quality of life and increases the cost of care, rate of hospitalization and ICU utilization.

**Methods:** Retrospective data collection from files of patients who passed away under medical oncology or palliative care departments. Then categorized to patient received therapy within the last 60 days before end of life or more than 60 days. The main variates are: Age. Disease site. date of diagnosis. Date of DNR order how many Lines of chemotherapy given. Date of last chemotherapy Date of Death.

**Results:** *Interim Analysis:* Mortality cases under medical and palliative oncology from January till September 2019 have been collected. Total of 78 cases, 47 females and 31 males were evaluated. The median age was 56 years, 49 patients (62.8%) received chemotherapy in the last 60 days of life. 18 pts (23%) received chemotherapy in the last 30 days of life. Median lines of chemotherapy were 2. Causes of death are to be evaluated as progressive disease or chemotherapy toxicity.

**Conclusion:** From this single center experience, we observe the over usage of systemic anticancer therapy towards end of life, regardless of the cancer type, age, gender or who prescribe the therapy. Considerable number of the study subjects had received only one line of systemic therapy and majority of them just received the first cycle.

## Incidence of Colorectal Cancer in Saudi Arabia: A Nationwide Analysis and Projection to 2030

Safy Othman^1^, Yasmine Zaatreh^1^, Aida Saad^1^, Omar Da’ar^2^, Mohammad Alkaiyat^3^, Abdul Rahman Jazieh^3^

^1^College of Medicine, Alfaisal University, Riyadh, KSA; ^2^King Saud Bin Abdulaziz University for Health Sciences, Ministry of National Guards, Riyadh, KSA; ^3^Department of Oncology, King Abdulaziz Medical City, Ministry of National Guard Health Affairs, Riyadh, KSA

**Introduction:** The aim of this project is to study the trends of colorectal cancer among Saudi Arabian patients estimated between the years of 1999 and 2014 and its relationship with determining factors such as nationality, gender, age, and the period of the incidence. In addition, this study postulated the forecast incidence of Colorectal Cancer from 2014 to 2030 in correspondence with Saudi Arabia’s vision.

**Methods:** Data was retrospectively collected from Saudi Cancer Registry (SCR), a nationwide record of colorectal cancer, and the number of new cases of colorectal cancer reported annually (1999– 2014) was stratified according to gender, age group, nationality, and year of diagnosis. Statistical analysis was carried out using the STATA package (Version 13, STATA Corporation, TX, USA).

**Results:** The statistical analysis provided in this study has shown that the prevalence of colorectal cancer in Saudi Arabia has increased from the years of 1999 through 2014 from 267 cases to 1760 cases, respectively,, with a male predominance in all age groups and a higher incidence with increasing ages particularly 50 years and above. With the steady elevation of the number of new cases diagnosed in this time period, this study predicts an increase of 93% in colorectal cancer incidence by 2030.

**Conclusion:** After a thorough study of the SCR colorectal records, we predict a steady rise of colorectal cancer among Saudis with male predominance by 2030.

## Outcome and Complications of Inferior Vena Cava Filter Insertion: Tertiary Referral Center Experience

Gmati GE, AlRasheed RF, Bukhary GA, Shaheen NA, Immanuel A, Alaskar AS,Salman RE

King Abdulaziz Medical City, Ministry of National Guards Health Affairs, Riyadh, KSA

**Introduction:** Pulmonary embolism (PE) remains a source of significant mortality and morbidity all over the world and in Saudi Arabia, the exact incidence remains unknown; however, deaths due to venous thromboembolism (VTE) and PE was estimated 1012% in hospitalized patients. While most patients are managed by oral anticoagulants; many risk factors preclude their use and recurrent PE remains a major risk. Inferior vena cava (IVC) filters has been introduced since 1969, many filter types were developed since then which had better outcomes and less complications. The aim of the study was to identify the indications and complications of IVC filter insertion as well as to estimate the success rate of IVC filter insertion performed at King Abdul-Aziz Medical City, Riyadh Saudi Arabia.

**Methods:** A retrospective cohort design was employed. The medical charts of patients who had IVC filter insertion from 2011–2016 were reviewed. The main referring departments for the procedure were internal medicine, medical oncology, orthopedics, and emergency medicine. Demographic variables, indications of insertion, outcomes, and complications of IVC filter insertion were collected. All patients underwent the procedure in the intervention radiology suit. Categorical variables were summarized as proportion and percentage. Continuous variables were summarized as mean and standard deviation. Data was analyzed using SAS version 9.2.

**Results:** Total of 411 patients were eligible based on the inclusion criteria. Male to female distribution was 61.07% to 38.93% respectively. Main indication for filter insertion was calculated based on the latest Society of International Radiology (SIR) guidelines. The most common indication of insertions was the contraindication to oral anticoagulants prescription in 131 (37.86%) patient followed by PE or DVT and transient inability to anticoagulate in 65 (18.79%) patients while 65 (18.79%) patients did not have a clear indication documented. The types of filters used were Optease filters in 308 (75%) patients, followed by Denali in 62 (15%) patients, and other less commonly used filters, for instance, Celect filters in 41 (10%) patients. Anatomically, 400 (97.32%) filter were inserted infrarenally, 9 (2.20%) filters were inserted suprarenally, and 2 (0.49%) were inserted in the common iliac vein. The rate of Insertion problems was 16.1% (n = 66). Filter migration was reported in 2 patients (0.49%). Retrieval was successful in 153 (84.97%) of patients who were followed up. IVC filter penetration occurred in 2 (0.49%) of our patients. No report of immediate complications in 344 (83.90%) patients. Immediate complications reported were in 67 (16.1%) patients, and the most common one was tilting in 56 (13.66%) patients. Mean duration of filter in situ was 91.91 days. Loss of follow up due to death unrelated to procedure occurred in 113 (27.90%) patients and in 87 (21.48%) patient follow up lost due to other reasons. The most common late complication was thrombosis in 38 (9.38%) patients. IVC thrombus complicated retrieval in 8 (1.97%) patients and failure to retrieve due to other comorbidities and implications occurred in 6 (1.48%) patients.

**Conclusion:** IVC filter placement in our institution is considered to be a safe procedure with minimal complications and high success rate of filter retrieval. A multicenter study is needed to compare the IVC insertion outcomes across facilities.

## Resource Use and Cost Estimate at End-of-life Care for Patients with Lung Cancer

Ali Alkhaibary^1^, Saud Al-Sarheed^1^; Mohammad Alkaiyat^2^; Omar B. Da’ar^1^; Abdul Rahman Jazieh^2^

^1^King Saud bin Abdulaziz University for Health Sciences, Riyadh, KSA; ^2^Department of Oncology, King Abdulaziz Medical City, Ministry of National Guard Health Affairs, Riyadh, KSA

**Introduction:** End-of-life care is a major concern to physicians managing cancer patients as significant healthcare resources can be utilized during this period. This study aims to evaluate the resources utilized at end-of-life care for patients diagnosed with lung cancer at a tertiary cancer facility.

**Methods:** This is a retrospective cohort study for patients diagnosed with lung cancer between the period of January to December2016 at King Abdulaziz Medical City, Riyadh, Saudi Arabia. Cost estimates of the clinical procedures, healthcare facilities, and resource usage were carried out using hospital and market information. All patients meeting the inclusion criteria and diagnosed with lung cancer were included. Differences in costs of various kinds of clinical and facility resource utilizations were performed for palliative and non-palliative care patients.

**Results:** Sixteen patients were included in the study with a mean (SD) age of 65.37 ± 13.40 years. Chest radiographs were the most frequent imaging modality, performed on average eight times at end-of-life. Basic chemistry screening and kidney function tests were the most frequent laboratory workups performed. The average CBC and chest radiograph costs between non-palliative and palliative referred patients differed significantly (p = 0.048 and 0.018, respectively), and they were eight and 40 times costlier in non-palliative patients.

**Conclusion:** Patients with lung cancer at end-of-life utilize significant healthcare resources. Non-palliative patients have significantly higher costs than palliative referred patients. Thus, it is important to emphasize on orderly resources allocation in order to provide optimal services for patients with lung cancer at their end-of-life.

## Improvement of Dental Referral Process for Oncology Patients on Bone Modifying Agents

Nafisa Abdelhafiez, Manal Humaid, Ahmed Al-Qudimat, Mohamad Alqahtani

Department of Oncology, King Abdulaziz Medical City, Ministry of National Guard Health Affairs, Riyadh, KSA

**Introduction:** Bone modifying agents are used widely in oncology. Patients treated with bisphosphonate or denosumab should undergo a dental examination with preventive dentistry prior to the initiation of therapy per standard of care to reduce the risk of osteonecrosis of the jaw (ONJ). This lack of standard has a significant negative impact on clinical outcomes and delay treatment. Therefore, decreases patient satisfaction. Data taken over three months shows great fluctuation from referral to clearance date.

**Methods:** The data was obtained for the following time intervals: from initial dental referral date to acceptance or refusal and from acceptance date till dental clearance.

**Results:** 143 patients were referred from adult medical oncology to dental department from May to July 2019. 21 patients were specifically referred for BMA clearance. Zero occurrence of ONJ among patients who received BMA. Referral pathway established with positive outcome achieved. Data monitoring still ongoing.

**Conclusion:** Close coordination between the treating physician and oral surgeon / a dental specialist will ensure that oncology patient will receive the appropriate dental care in timely fashion.

## Use of Complementary/Alternative Medicine in Children with Cancer in Saudi Arabia: A Tertiary Care Center Experience

Reem Al Sudairy, Ali Omari, Talal Harbi, Khalid Jamaan, Mohammed Essa, Abdulrahman Al Sultan, Enas Bashir, Reem Abdellatif, Nagham Sheblaq, Mohammad Alkaiyat and Abdul Rahman Jazieh

King Abdulaziz Medical City, Ministry of National Guard Health Affairs, Riyadh, KSA

**Introduction:** The use of complementary and alternative medicine (CAM) in children with cancer has been documented in the literature worldwide, but there is scarce information about this subject from Saudi Arabia. This study aims to explore the prevalence and pattern of CAM use in this population.

**Methods:** This is a cross-sectional descriptive study which included pediatric cancer patients (up to age 16 years) who are treated at King Abdullah Specialist Children Hospital in Riyadh, Saudi Arabia. An interview-administered questionnaire was used. CAM use was divided into dietary and non-dietary forms.

**Results:** A total of 211 patients with median age of 6 years were included (0.1–16) in the study. Parents were the primary care givers in 96.7% of patients. Around 70% of parents were high school and university graduates with monthly income of more than 6000 SR. There were 139 (66%) patients with leukemia and lymphoma, 56 patients (26.5%) with solid tumors, and 16 patients (7.5%) with brain tumors. Parents of 87 patients (41.2%) thought that the cause of the disease was “envy” by others. A total of 184 (87.2%) patients used non-dietary CAM like Quran Recitation and Rugya (86.8%), applying olive oil as lotion (47.9%), and showering with Zamzam water (17.9%). Dietary supplements were used by 60 patients (28.4%) including drinking Zamzam water (31.1%), honey (19.5%), and black seed (10%). Drinking camel urine was reported by 3.7% and using unknown source of herbal mixtures by 1.6% of dietary CAM users. Physicians were informed about the use of non-dietary CAM by 18.5% of parents while 30% of parents who used dietary CAM discussed it with their physicians. Delay of medical treatment was decided by 10% of parents while using CAM. Reasons for using CAM were to treat cancer as part of their religious beliefs, improve the psychological status in 9.5%, and to improve the immune system in 7.9%.

**Conclusion:** Our study revealed that CAM is widely used among pediatric patients with cancer. Most of it had religious background as well as traditional dietary habits, highlighting the need for the medical team to discuss closely the use of CAM with their patients upfront in order to educate them about the benefits and harmful effects of such practices.

## Measuring the Burnout Level among Oncology Physicians: Baseline Data for a Quality Improvement Project

Nashmia Joudallah Mutiri, Mona Shami, Mohammad Alkaiyat, Manar Mazroua, Faisal Al-Hamdan, Tabrez Pasha, Abdul Rahman Jazieh

King Abdulaziz Medical City, Ministry of National Guard Health Affairs, Riyadh, KSA

**Introduction:** Burnout is a syndrome characterized by emotional exhaustion that results in depersonalization and decreased personal accomplishment at work. The emotionally exhausted clinician is overwhelmed by work to the point of feeling fatigued, unable to face the demands of the job, and unable to engage with others. Our project aimed at determining the prevalence of burnout among our oncology physicians.

**Methods:** We used a cross-sectional data collection technique to measure the burnout level (baseline) among doctors in the Department of Adult Medical Oncology, Maslach Burnout Inventory (MBI) was used to assess the occupational professional burnout, assess and validate the three-dimensional structure of burnout, and to understand the nature of burnout in order to develop effective interventions among doctors in the department.

**Results:** A total of 60 doctors were interviewed and their data were analyzed. 41 (68.3%) were male, the majority are married 51 (85%) and more than half of the sample 36 (60%) have been working in the department for equal or less than 5 years. In the first section in the MBI, which focuses on the burnout (or depressive anxiety syndrome), data showed that only 3 doctors (5%) have high-level burnout. Section B of the MBI, which focused on depersonalization, showed that only 19 doctors (31.7%) have high-level burnout, while the third section, which focuses on personal achievement, showed that only 14 doctors (23.3%) have high-level burnout in this domain. The accumulative analysis showed that 27 (45%) of doctors are having occupational burnout. Most of the staff reported workload as the main contributed factor to develop burnout.

**Conclusion:** The burnout among our doctors is high, some doctors showed burnout in more than one dimension on MBI. This high level of burnout may affect the doctors on the emotional and physical which will negatively impact their performance at work. In the near future, systematic sets of intervention are needed to help doctors overcome/avoid burnout.

## Prevalence and Patterns of Use of Complementary and Alternative Medicine by Patients with Cancer in Saudi Arabia

Khadega Abuelgasim^1^, Mohammad Alkaiyat^1^, Omar B. Da’ar^2^, Abdul Rahman Jazieh^1^

^1^Department of Oncology, King Abdulaziz Medical City, Ministry of National Guard Health Affairs, Riyadh, KSA; ^2^King Saud bin Abdulaziz University for Health Sciences, Ministry of National Guards Health Affairs, Riyadh, KSA

**Introduction:** The use of complementary and alternative medicine (CAM) is common among patients. Yet, there is limited evidence of the extent to which this has changed over and its association with socioeconomic and clinical factors of cancer patients. This study aimed to: (1) assess whether the use of CAM has changed over time; (2) the relationship between use of CAM and socioeconomic and clinical factors of patients; and (3) how conventional therapies affect the use of CAM.

**Methods:** A two-phase cross-sectional study of oncology patients in oncology wards and outpatient clinics at KAMC using face-to-face interviews.

**Results:** A total of 464 patients was analyzed in phase I (2006–2008), while 952 patients were analyzed in phase two (2009–2018). Together, there were 865 (61.1%) females and the median age was 56 years (11–98). The majority (96.7%) of the patients sampled during phase I used CAM compared with 78.9% among patients sampled during phase II. A total of 1016 (85%) of all the sampled patients in the two phases used some type of dietary supplement as CAM (e.g. camel products and black seeds). Seventeen percent (17%) of patients in phase I attributed their improvement to CAM use compared to 4% in phase II. However, 7.6% of patients in phase I attributed their improvement to medical treatment compared with 29% in phase II. Nearly two-fifths (43%) of the patients incurred some monthly cost during phase 2 compared with 74% during phase 1. Being a female cancer patient, the odds of using dietary supplementary CAM (versus not using DS) increase by a factor of 1.87. Being a married cancer patient, the odds of using dietary supplementary CAM (versus not using DS) increase by a factor of 1.82. Having CAM use with the doctor, the odds of using dietary supplementary CAM (versus not using DS) increase by a factor of 3.1. Having a monthly cost for use of CAM is associated with the odds of using dietary supplementary CAM (versus not using DS) increasing by a factor of 15.

**Conclusion:** Cancer patients are increasingly using CAM and the concurrent conventional therapies appear not to act as deterrents. Socioeconomic factors appear to explain more this phenomenon than do clinic factors. While the use of CAM may be beneficial, it can also exacerbate other underlying clinical issues, especially when substituted for conventional medical intervention and when some dietary supplements are linked to other opportunistic diseases.

## Metabolic Syndrome and Risk of Developing Colorectal Cancer in Saudi Arabia

Shurouq Alqahtani, Hoda Jradi

King Saud bin Abdulaziz University for Health Sciences, Ministry of National Guards Health Affairs, Riyadh, KSA

**Introduction:** Colorectal cancer (CRC) is the third leading cause of mortality worldwide and second most common cancer in both sexes in Saudi Arabia. The relationship between metabolic syndrome (Mets) and CRC is still under investigation and an area of questioning regarding to what extent each component accounts for the positive and independent association. This study aims to examine the association between Mets components and CRC incidence among Saudis. Also, it aims to emphasize the role of public health in early detection of associated risk factors.

**Methods:** This is a matched case control study conducted in tertiary hospital in Riyadh. Data were retrieved from hospital medical records; all patients were recruited from endoscopy unit between 2018 to 2019. Out of 1,843 patients, a total of 127 confirmed CRC cases were matched with 127 cancer free controls. Descriptive statistics were reported, and conditional logistic regression analysis was conducted to assess the likelihood of study variables related to Mets as being a significant risk factor for CRC. The components of Mets among cases and controls were measured based on the National Cholesterol Education Program Adults Treatment Panel III (NCEP ATP III) criteria.

**Results:** Overall this study identified that 31.49% of CRC patients met Mets criteria in comparison to only 18.11% of controls. Controlling for all other factors, history of other cancer (OR = 3.41; 95% CI: 1.14–10.27) and hypertriglyceridemia (OR = 2.07; 95% CI: 1.02–4.18) were the remaining independently and significantly associated factors with CRC in multivariate conditional logistic regression.

**Conclusion:** Findings indicate that the risk of developing colorectal cancer is almost double in individuals with Mets. Hypertriglyceridemia was identified as a significant and independent risk factor in CRC patients. The importance of CRC screening program among patients with history of other cancer types needs for further analytical and multicentric studies.

## Satisfaction Among Cancer Patients: Findings from Tertiary Hospital in Riyadh

Alanood Alharbi, Maali Alrashed, Mishaal Alshoaibe, Hoda Jradi, Mohammad Alkaiyat, Abdul Rahman Jazieh

King Saud bin Abdulaziz University for Health Sciences, Ministry of National Guards Health Affairs, Riyadh, KSA

**Introduction:** Patients’ satisfaction with their cancer care is a critical issue in assessing the quality of cancer care. In Saudi Arabia, cancer patients’ satisfaction at tertiary care hospitals of Riyadh city has not been reported. This study aimed to assess the level of satisfaction with cancer care and related factors at a tertiary care teaching hospital in Riyadh City, Saudi Arabia.

**Methods:** Data were collected from patients based on a validated questionnaire (Quality Health/National Health Service, UK). A total of 266 cancer patients aged (19–85 years) were sampled. Descriptive statistics were reported and regression analysis to estimate the likelihood of satisfaction among this sample.

**Results:** The majority of respondents were females (74%), age 54–62 years, with employment status as homemakers. The majority of the patients were satisfied with overall care provided and reported that their care needs were met. However, others were not satisfied with the given amount of information given about their condition and the treatment. Main reports of satisfaction were for: the time they were seen; sensitivity of breaking bad news; involvement in treatment and care; satisfaction with the hospital staff for doing everything they could to help control their pain; and given enough emotional support from hospital staff.

**Conclusion:** Most of the surveyed patients were satisfied with the cancer care. However, there were several factors that were reported as unsatisfactory such as the satisfaction with the amount of information given about condition and the treatment. Future research is needed to assess the health system and sociocultural determinants of patient satisfaction with cancer care in the country as a platform for policy makers and cancer guidelines formulation.

## Health-Related Quality of Life and Perceived Social Support Among Patients Receiving Palliative Cancer Care in Saudi Arabia

Manal Banaser^1^, Sami Alshammari^2^

^1^Nursing Affairs General Department, Ministry of National Guards Health Affairs, Riyadh, KSA; ^2^King Fahd Medical City, Riyadh, KSA

**Introduction:** Palliative cancer care is becoming a core aspect of cancer treatment. Cancer patients are increasingly demanding a multidimensional approach to care, including physical, emotional, psychological, and spiritual dimensions. Health-related quality of life (HRQL) is essential to the attainment of these functions. At the end of life care, patients require improved quality of life through palliative care, involving symptoms control, family support, and satisfaction, as well as a patients’ sense of purpose and meaning. This study aims to describe HRQL, identify factors associated with HRQL physical and mental health domains, and explore the perceived social supports for cancer palliative patients in Saudi Arabia.

**Methods:** A cross-sectional study design was used. The validated European Organization for Research and Treatment of Cancer, EORTC QLQ-15 palliative care scale, and the Perceived Social Support Scale (PSSS) were used. Additional sociodemographic and health status data were collected, including age, gender, education level, residency area, social status, time of diagnosis, and cancer type. A convenience sample of 301 adult palliative cancer patients from two Saudi regional cancer centers in Riyadh was included. Data were analyzed using SPSS descriptive statistics and correlational analysis.

**Results:** Results indicates that overall quality of life showed a significant positive correlation with perceived family and friend support, sub-factors of perceived social support. Regression analysis showed that the overall model experienced 69.0% of the variance for global health statutes with F (4, 7) = 7.149 p < 0.01. Physical functioning, emotional functioning, and family support were found to be significant predictors of global health status, whereas diagnosis time was found to be a significant predictor of global health status. Family and Friend support were found to be significant positive predictors of emotional functioning, whereas education level and diagnosis time were found to be significant negative predictors of physical functioning. However, diagnosis time was found to be a significant positive predictor of emotional functioning.

**Conclusion:** The inpatient and outpatient treatment can vary at different stages and in different areas, family and friend support has been highlighted as necessary in this context. Physical and emotional variables have been highlighted in older age (geriatric) patients as they may have debilitating diseases that can limit their functioning hence support the case for more palliative care.

## Immunotherapy Prescribing Patterns in the Oncology Department of a Tertiary Care Hospital: A Retrospective Study in Riyadh, Saudi Arabia

Menyfah Q. Alanazi, Hana Al Abdulkarim, Sami Al-Ajlan, Abdul Rahman Jazieh

King Saud bin Abdulaziz University for Health Sciences, Ministry of National Guards Health Affairs, Riyadh, KSA

**Introduction:** Pembrolizumab, nivolumab, and atezolizumab are US Food and Drug Administration (FDA)-approved immunotherapy agents for specific indications. These drugs are extremely costly to the healthcare system, payers, and ultimately, patients, and little is known about their utilization for non-FDA labeled indications (off-label). The aim of this study was to assess the prevalence of off-label Immunotherapy indication and evaluate the utilization patterns and economic impact of Immunotherapy at King Abdulaziz Medical City (KAMC), Riyadh, Saudi Arabia.

**Methods:** A retrospective cross-sectional descriptive study was conducted over a 3-years period at KAMC-R in Saudi Arabia. Data on demographics, clinical characteristics, and diagnosis were collected.

**Results:** During the study period, there were estimated 285,139 drug prescriptions from oncology pharmacies, of which, 1,285 were Immunotherapy prescriptions. The prevalence of prescribed Immunotherapy prescriptions was 0.45%. Of the prescriptions, (3.16%) were prescribed for children (< 18 years), while (96.8%) were prescribed for adult patients; thus, immunotherapy drugs were prescribed for adults significantly more than children (p = 0.001). The majority of patients were males (67.89%) with ratio 2.1:1, males to females (median age 50.5 ± 54.45 years, range 12–89 years). Patients were treated mainly for lung cancer and hepatocellular carcinoma (27.89 and 16.84%, respectively). Other types of cancer were observed at lower rates, including renal cell carcinoma (8.42%), Hodgkin lymphoma (7.89%), and malignant melanoma (7.89%). Prevalence of off-label indication was (9.5%). The most common off-label indications were diffuse large B-cell lymphoma, representing prostate cancer. The proportion of utilization of nivolumab outside the Pharmaceutical and Therapeutic Committee (P&T)-approved indication represented 72.9% of the total patients during study period. The average cost of immunotherapy prescriptions prescribed was $1,922,509.15 ± $198,900.00 USD with costs ranging from SAR 3.1 to 523.5 ($11,604.00 to $141,110.00 USD).

**Conclusion:** The results revealed a significant level of off-label indication uses of immunotherapy in KAMC. Generally, it seems that there is a need for improving the prescribing patterns of nivolumab in KAMC. Restriction policies, periodic assessment and monitoring and multidisciplinary efforts to control and monitor nivolumab usage are urgently required.

## Do Cancer Patients’ Demographics and Clinical Conditions, Reason for CAM use, Sources of Information, and Complementary Therapy with Healthcare Providers Affect the Likelihood of Dietary Supplement CAM use?

Omar B. Da’ar, Mohammad Alkaiyat, Khadeja A. Abuelgasim, Abdul Rahman Jazieh

King Saud bin Abdulaziz University for Health Sciences, Ministry of National Guards Health Affairs, Riyadh, KSA

**Introduction:** While complementary and alternative medicine (CAM) use among oncology patients is common, there is limited evidence in both scope and methodology of what influences the likelihood of dietary supplement CAM use. This study examined the direction and magnitude of the effects of patients’ demographics and clinical conditions, reason for CAM use, sources of information, and complementary therapy with healthcare providers on the likelihood of dietary supplement CAM use.

**Methods:** A cross-sectional study of 952 oncology patients in oncology wards and outpatient clinics at KAMC using face-to-face interviews between 2016–2018. Using a probit model, we estimated the effect of cancer patients’ demographics and clinical conditions, reason for CAM use, sources of information of the use of CAM, and complementary therapy with healthcare providers (e.g., doctor and nurse) on the likelihood of dietary supplement.

**Results:** Overall, 79% of the 952 patient interviewed used CAM and 69% of them used dietary supplements. Nearly three-fifths (61%) were female. The median age was 56 (range 17–98). Majority of the patients (81%) had solid tumor. The results suggest that females were associated with more use of dietary supplement CAM use (p < 0.001) compared with male patients. Compared with solid tumor, hematological malignancy was associated with more use of dietary supplement (p = 0.009), while other cancer types such as stem cell transplant, and brain tumor were not significant. Patients’ complementary therapy with a doctor and not a nurse was associated with more use of dietary supplement CAM use (p = 0.001). As sources of information on CAM use, social beliefs (p <0.001), contact with herbal medicine practitioners (p = 0.033), and television and/or internet (p <0.001) were associated with more use of dietary supplement. Reason for CAM use such as religious belief (p <0.001) and improvement of mood level (p = 0.021) were associated with more use of dietary supplement CAM use.

**Conclusion:** Save for gender, results suggest socioeconomic and demographic factors such as education level, marital status, age, employment status, and income were not associated with the use of dietary CAM. However, clinical conditions, reason for CAM use, sources of information of the use of CAM, and complementary therapy with healthcare providers appeared to influence on dietary use of CAM. These findings may have implications for conventional medical intervention.

